# Prevalence of smoking and its associated factors with smoking among elderly smokers in Malaysia: findings from a nationwide population-based study

**DOI:** 10.1186/s12971-016-0073-z

**Published:** 2016-03-21

**Authors:** K. H. Lim, K. Jasvindar, S. M. Cheong, B. K. Ho, H. L. Lim, C. H. Teh, K. J. Lau, A. Suthahar, D. Ambigga

**Affiliations:** Institute for Medical Research, Jalan Pahang, 50590 Kuala Lumpur, Malaysia; Institute for Public Health, Jalan Bangsar, 50588 Kuala Lumpur, Malaysia; Klang Health Department, Bandar Botanic Clinic, 41200 Klang, Selangor Malaysia; Melaka Manipal Medical College, Jalan Pengkalan Batu, Bukit Baru, 75150 Melaka Malaysia; School of Medical Science, Universiti Sains Malaysia, Kuang Kerian, 15000 Kelantan, Malaysia; Faculty of Medicine, University Teknologi Mara, Sg Buloh, 47000 Selangor, Malaysia; Faculty of Medicine and Defence Health, University of Defence, Kem Sg. Besi, 57000 Kuala Lumpur, Malaysia

**Keywords:** Elderly, Prevalence of smoking, Population-based study, Malaysian

## Abstract

**Background:**

The determination of smoking prevalence and its associated factors among the elderly could provide evidence-based findings to guide the planning and implementation of policy in order to will help in reducing the morbidity and mortality of smoking-related diseases, thus increase their quality of life. This paper describes the rate of smoking and identifies the factor(s) associated with smoking among the elderly in Malaysia.

**Methods:**

A representative sample of 2674 respondents was obtained via a two-stage sampling method in proportion to population size. Face-to-face interviews were conducted using a set of standardized validated questionnaire. Data was weighted by taking into consideration the complex sampling design and non-response rate prior to data analysis. Univariable and multivariable logistic regression were used to determine the factor/s associated with smoking.

**Results:**

The prevalence of non-smokers, ex-smokers and current smokers among Malaysians aged 60 years and above were 36.3 % (95 % CI = 32.7–39.8), 24.4 % (95 % CI = 21.2–27.5) and 11.9 % (95 % CI = 9.5–14.3), respectively. Current smokers were significantly more prevalent in men (28.1 %) than in women (2.9 %), but the prevalence declined with advancing age, higher educational attainment, and among respondents with known diabetes, hypertension and hypercholesterolemia. Multivariable analysis revealed that males (aOR, 18.6, 95 % CI 10.9-31.9) and other Bumiputras (aOR 2.58, 95 % CI 1.29-5.15) were more likely to smoke. in addition, elderly with lower educational attainment (aOR, 1.70, 95 % CI 1.24-7.41) and those without/unknown hypertension also reported higher likelihood to be current smokers (aOR 1.98, 95 % CI 1.35-2.83). However, there were no significant associations between respondents with no/unknown diabetes or hypercholesterolemia with smoking.

**Conclusions:**

In short, smoking is common among elderly men in Malaysia. Therefore, intervention programs should integrate the present findings to reduce the smoking rate and increase the smoking cessation rate among the elderly in Malaysia and subsequently to reduce the burden of smoking-related disease.

## Background

Advancements in healthcare have contributed to a longer lifespan among the Malaysian population, which had increased from 68 years in 1980 to 75 years in 2013 [1]. It is anticipated that the elderly population in the country will increase from 6.0 % (1.5 millions people) in 2000 to 11.5 % (3.4 million) by 2020 [2]. In view of the changing in the Malaysian population structure, the Malaysian government has taken pro-active measures by formulating the elderly policy to create dignified and successful aging among the Malaysian elderly in Malaysia and to ensure they can lead a normal life in their old age [3]. The objectives of the policy were in line with the “successful aging” concept by John Rowe and Kahn [4] and World Health Organization [5], which define normal living during old age as having a low risk of disabilities, diseases, and disabilities due to diseases as well as having higher physical and mental functions.

However, smoking-related morbidity and mortality have been identified as one of the factors that jeopardised the “successful aging” concept. Many studies had demonstrated that elderly people who smoked had 10 years shorter lifespan and poorer health conditions than those who did not smoke [6]. Besides, men and women who ceased smoking at the age of 65 gained 1.4–2.0 and 2.7–3.4 years of life, respectively; and those who stopped smoking at 70 year-old had an increased survival of about 20 % [7]. In addition, it had also been shown that the circulatory function improved immediately after smoking cessation and the lungs begin to repair the damages within 1 year. The risk of having heart disease and stroke had also reduced by 50 % [8]. A national study in Singapore revealed a reduction in the risk for total mortality, specifically for lung cancer mortality, within 5 years of smoking cessation [9]. In order to realise the goal of successful aging among the Malaysian elderly, factors contributing to elderly smoking should be identified for targeted and specially tailored interventional programmes.

A Malaysian nationwide study revealed that the smoking prevalence was 39.4 and 19.8 % in year 2002 and 2006, respectively [10, 11], in which 34.0 % of them attempted to quit and 67.7 % planned to quit, respectively [12]. The Ministry of Health Malaysia have launched several initiatives to decrease the smoking prevalence by intensifying the health promotion activities, increasing the price of tobacco products, introducing more non-smoking areas, increasing the number of smoking cessation clinics in primary health care centred, to promote smoking cessation amongst the smoking population in Malaysia, including the elderly sub-population [13].

A number of studies that investigated smoking among elderly had unequivocally demonstrated that respondents of advancing age [10] and higher educational attainment [14–17], being married [18] or working in professional professions [14, 15] were less likely to smoke. In Malaysia, studies on smoking have been started since the last 30 years, however the those studies mainly focus only on the adolescents and adults, and little attention was given towards the elderly. To our knowledge, only two studies on elderly smoking were reported for the past 10 years. However, those studies on investigated the smoking prevalence, knowledge and attitude of the elderly toward smoking [10, 19], but factors associated with smoking have not been given due attention. Since the factors of smoking among elderly had been reported to be varied from the general population in terms of smoking duration, health status, resistance to quit smoking, awareness on the potential harm of smoking and overestimation on the benefit of smoking [20, 21] as well as limited knowledge pertaining to elderly smoking in Malaysia, it is therefore timely to address these existing gaps in knowledge. It is imperative to determine the current prevalence and factors associated with smoking among a representative sample of elderly in Malaysia in order to provide evidence-based recommendations to policy-makers for planning, improvement and implementation of policies pertaining to prevention of smoking among the elderly populations for better health conditions and successful aging. Reduced smoking-related morbidity among elderly could definitely reduce the government economic burden in the health care sector and foster our national vision to become a develop country by 2020.

## Methods

The data was extracted from a cross-sectional national study, the National Health and Morbidity Survey 2011 (NHMS 2011). A representative sample of Malaysian population was selected using a two-stage stratified sampling proportionate to the population. The first stage stratification composed of the 15 states in Malaysia and the second stage stratification was by urban/rural locality for each state. The primary sampling unit (PSU) or enumeration block is the an artificial geographically-continuous area with identified boundaries created by the Department of Statistic Malaysia which contains 80 to 120 living quarters (LQs) in each PSU, and these LQs were the secondary sampling unit. Twelve LQs were randomly selected from each selected EBs and all eligible households in the selected LQs were selected for face-to-face interview by trained research assistants. In total, 794 EBs (484 from urban areas and 310 from rural areas) composed of 9,528 households were recruited into the survey. Detailed explanation of the sampling method was described by Fadhli et al. (2013) [22].

### Instrument and measurement

Questionnaire was developed by a panel of experts consist of Public Health Specialists and health education officers. The questionnaire consisted of several components, namely social demography, risk behaviors (smoking, alcohol consumption), current health status (presence of diabetes, hypertension or hypercholesterolemia). The instrument was first developed in English and was translated into few other languages (Bahasa Malaysia, Mandarin and Tamil) used by the major ethnic groups in Malaysia. The instrument was pre-tested among respondents in Bangsar, Kuala Lumpur. Data collection proceeded for 4 months, from April to the July 2011.

Written consent was obtained from each respondent prior to the interview by trained research assistants. To ensure a high response rate, unoccupied households were revisited up to three times. The present study was approved by the Medical Research Ethics Committee, Ministry of Health, Malaysia.

The dependent variable. Smoking status was evaluated by the following items: “Have you ever smoked shisha, cigarettes, cigars, pipes, etc?” and “Do you currently smoke?”. Respondents who answered “Yes” to both questions were categorised as “current smokers” whilst those who answered “Yes” to the first question and “No” to the second question were categorised as “Ex-smokers”. Respondents who answered “No” to both questions were categorised as “Never smoked”. In our analysis, ex-smokers, ever smokers and never smokers were combined and constituted the non-smokers category.

Key socio-demographic variables included age, gender, marital status, highest education level attained, occupation, residential locality and household income level. Education attainment was categorized into four levels as no formal education, primary education (1–6 years), secondary education (7–12 years), and tertiary education (more than 12 years and enrolled in university). For marital status, respondents were categorised as married or single/widow/widower/separated. Data on monthly household income was obtained using an open-ended question asking for the exact income which was later categorized into three categories: less than RM 2000, RM 2000–2999 and RM 3000 and above. The age of respondents was divided into four categories as 60–64 year-old, 65–69 year-old, 70–74 year-old and 75 years and above).

### Measurement of hypertension, diabetes and hypercholesterolemia

#### Hypertension

Hypertension status of respondents were determined by the question “Have your ever been told by a doctor or medical assistant that you have hypertension?”. Those who answered “Yes” were classified as “known hypertension”. On the other hand, respondents who answered “No” would have their blood pressure measured by trained nurses using a pre-calibrated digital blood pressure device (OMRON HEM-907.) according to both the American Association for the Advancement of Medical Instrumentation (AAMI) and British Hypertension Society (BHS) protocols for accuracy for a non-invasive blood pressure monitoring device using single observer readings [23]. Respondents who perceived that they did not have hypertension but had a SBP of 140 mmHg or more and/or DBP of 90 mmHg or more [24] were categorised as “undiagnosed hypertension”. The measurement was taken in twice in an interval of 10 min and the average readings were used in subsequent data analysis.

#### Diabetes

Respondents were asked to fast for at least 6 h for the measurement of fasting blood glucose level using a validated CardioChek PA Analyser [25] via finger-prick test by trained nurses. “Known diabetes” is defined as self-report of being told they have diabetes by a doctor or medical assistant; a respondent was classified as having ‘undiagnosed diabetes’ when the respondent was not known to have diabetes and had a fasting capillary blood glucose (FBG) of 6.1 mmol/L or more or non-fasting blood glucose of more than 11.1 mmol/L [26].

#### Hypercholesterolemia

Hypercholesterolemia status of respondents were determined by the question “Have your ever been told by a doctor or medical assistant that you have hypercholesterolemia?’. Respondents who answered “Yes” were classified as “Known Hypercholesterolemia”, whilst respondents who answered “No” would have their blood tested by trained nurses using a pre-calibrated cardio-check meter. Respondent with blood cholesterol level higher than 5.2 mmol/l were classified as undiagnosed hypercholesterolemia [27].

### Statistical analyses

Data analysis was performed using SPSS version 20 and STATA version 11. Data was weighted by taking into consideration the complex sampling design and non-response rate. Descriptive statistics were used to illustrate the social demographic characteristics of respondents. Multivariable logistic regression was used to determine the real effect of each independent variable on smoking after the effects of other variables were controlled for. Finally, the fitness of the final model of factors was checked by STATA version 11 using the modified Hosmer-Lemeshow Goodness of Fit test for complex sampling [28]. A non-significant *p*-value (>0.05) meant that the model had a good fit. Tests for possible two-way interactions in the final custom model performed showed that no significant interactions were present. The data was presented in 95 % confidence intervals without *p* values as the large sample size of the present study could generate significant results even if statistical differences or associations were small. All statistical analyses were carried out at 95 % confidence interval.

## Results

A total of 2674 elderly responded to the study, in which 51.5 % of them were females, approximately two-third of them were from urban areas (65 %) and married (67.9 %) but only 5.5 % of them were of tertiary educational level. and The distribution of the sample population by ethnicity was almost similar with that of the Malaysian general population. The mean age of the respondents was 68.7 year-old. The prevalence of current smokers among males was approximately 10 times higher than females (28.1 %, 95 % CI 24.8-31.6 vs 2.9 %, 95 % CI 1.9-4.4), whilst the proportions of current smokers among Malays (19.7 %, 95 % CI 17.1-22.6) and other Bumiputras in Sabah and Sarawak (21.3 %, 95 % CI 13.7-31.6) were significantly higher than that of Chinese (9,0 %, 95 % CI 6.9-11.7) and Indians (7.1 %, 95 % CI 3.8-14.3). Respondents who were younger, married, had low level of education attainment or from the rural areas, had higher prevalence of current smokers as compared to their counterparts who were older, single/widow/widower/separated, had higher education attainment or from the urban areas (Table 1).

**Table 1:** Social demography and smoking status variables among elderly aged 60 years and above in Malaysia

Variable	Non smoker				Ex-smoker				Current smoker			
	N	n	%	95 CI	N	n	%	95 CI	N	n	%	95 CI
Gender												
Male	483861	576	48.7	44.8-52.6	230512	307	23.2	20.2-26.5	279034	395	28.1	24.8-31.6
Female	980332	1355	93.7	91.9-95.1	35855	62	3.4	2.5-4.7	30552	44	2.9	1.9-4.4
Ethnic												
Malay	857583	969	65.8	62.8-68.7	144308	229	14.4	12.3-16.9	196808	302	19.7	17.1-22.6
Chinese	601387	633	79.9	76.1-83.1	84642	81	11.2	8.7-14.4	67637	77	9.0	6.9-11.7
Indian	100772	177	85.0	76.9-90.6	9398	12	7.9	4.0-12.2	8424	14	7.1	3.8-14.3
Others Bumiputra	98016	127	61.4	52.3-69.8	27593	42	17.3	12.0-24.3	34046	36	21.3	13.7-31.6
Age group												
60–64	553099	701	72.3	68.7-75.6	81248	108	10.6	8.5-13.2	131071	180	17.1	14.2-20.3
65–69	356419	467	72.3	67.8-76.4	62668	92	12.8	9.9-16.3	73698	109	14.9	11.8-18.7
70–74	261724	372	71.6	66.2-76.4	51910	81	14.2	10.8-18.3	51991	80	14.2	10.4-19.1
> 75	29292	391	70.4	63.4-74.9	70340	88	16.9	13.3-21.3	52826	71	12.7	9.3-17.1
Income level												
< RM 1000	515345	737	69.8	66.2-73.2	103963	145	13.6	11.1-16.4	127267	177	16.6	13.7-20.6
RM 1000-1999	274426	388	73.3	68.8-77.4	44529	71	11.9	8.8-15.9	55426	98	14.8	11.7-19.6
RM 2000-2999	212690	268	72.2	66.5-77.8	33326	54	11.4	8.3-15.5	47250	72	17.4	12.8-23.1
> RM 3000	131672	177	64.4	56.2-71.8	31007	34	15.2	10.0-22.3	41793	48	20.4	13.7-29.3
Marital status												
Married	910293	1162	65.7	62.7-68.6	217695	293	15.7	13.7-18.0	256896	356	18.8	16.1-21.3
Single	553901	769	84.5	81.3-87.3	48672	76	7.4	5.6-9.8	52691	84	8.0	6.1-10.6
Locality												
Urban	993586	1027	75.1	72.3-77.8	162279	173	12.3	10.2-14.6	166714	172	12.6	10.4-15.2
Rural	470608	904	65.6	62.3-68.7	104088	196	14.5	12.3-17.0	142873	268	19.9	17.2-23.0
Education attainment												
Not schooling	408497	625	77.4	73.6-80.8	53329	86	10.1	7.9-12.9	65793	99	12.5	9.8-15.8
Primary	704132	915	70.0	66,8-72.9	130919	188	13.0	11.0-15.3	171479	255	17.0	14.7-19.7
Secondary	257245	282	70.6	64.1-76.4	51709	65	14.2	10.4-19.1	55362	67	15.2	10.7-21.1
Tertiary	70785	81	64.9	53.7-74.8	25985	24	23.8	15.2-35.2	12264	12	11.2	5.4-21.8
Hypertension												
Known	623911	836	77.6	74.3-80.8	107643	142	13.4	11.1-16.1	70702	96	8.8	6.8-11.4
No/not known	832834	1084	67.8	63.9-70.5	158724	227	12.9	11.0-15.1	237018	340	19.3	16.8-22.4
Hypochloterolemia												
Known	329345	402	75.5	70.2-80.1	66980	89	15.4	11.7-19.9	39757	43	9.1	6.1-13.4
No/not known	1112136	1502	70.6	68.2-72.9	195862	275	12.4	10.8-14.3	267369	392	17.0	14.9-19.2
Diabetes												
Known	335162	467	74.6	69.9-77.8	73449	97	16.4	10.7-20.8	40575	60	9.0	6.6-12.2
No/not known	1111730	1443	71.2	68.6-73.6	189932	267	12.2	10.5-14.1	260500	368	16.7	14.5-19.1

Multivariable logistic analysis revealed that males were more likely to smoke compared to females (aOR 18.63, 95 % CI 10.9-31.9), the elderly of Malay and other Bumiputra descents were more likely to smoke (Malay, aOR 2.57, 95 % CI 1.67-3.96; other Bumiputras, aOR 2.58, 95 % CI 1.29-5.13) as compared to their Chinese counterparts. In addition, respondents who had lower education attainment (no formal education, aOR 3.10, 95 % CI 1.24-7.41), with undiagnosed hypertension or non-hypertensive respondents (aOR 1.98, 95 % CI 1.35-2.83) were more likely to smoke than their reference counterparts (Table 2).

**Table 2:** Multivariable analysis of factors associated with smoking among the elderly in Malaysia

Variable	Crude Odd Ratio	Adjusted Odd Ratio^a^	95 CI
Gender			
Male	12.99(8.43-20.21)	18.63	10.9-31.9
Female	1	1	
Ethnic			
Malay	2.49(1.76-3.53)	2.57	1.67-3.96
Chinese	1	1	
Indian	0.78(0.35-1.71)	1.04	0.46-2.42
Others Bumiputra	2.75(1.57-4.81)	2.58	1.29-5.15
Age group (years)			
60–64	1.42(0.95-2.12)	0.99	0.63-1.58
65–69	1.21(0.78-1.87)	0.91	0.56-1.47
70–74	1.14(0.71-1.84)	0.93	0.56-1.53
> 75	1	1	
Income level			
< RM 1000	1.32(0.91-1.91)	1.12	0.72-1.74
RM 1000-1999	1.15(0.76-1.73)	0.93	0.58-1.50
RM 2000-2999	1.27(0.80-2.02)	0.98	0.59-1.66
> RM 3000	1	1	
Marital status			
Married	1	1	
Single/widow/er	0.38(0.27-0.54)	0.83	0.52-1.32
Locality			
Urban	1	1	
Rural	1.72(1.31-3.27)	1.13	0.80-1.54
Education attainment			
Not schooling	1.12(0.49-2.59)	3.10	1.24-7.41
Primary	1.62(0.72-3.65)	1.47	0.90-4.31
Secondary	1.42(0.59-3.41)	1.39	0.61-3.12
Tertiary	1	1	
Hypertension			
Known	1	1	
No/not known	2.47(1.77-3.45)	1.98	1.35-2.83
Hypercholesterolemia			
Known	1	1	
No/not known	2.03(1.28-3.24)	1.26	0.76-2.68
Diabetes			
Known	1	1	
No/not known	2.05(1.39-2.93)	1.56	0.98-2.50

^a^ *Multivariate Logistic Regression analyses adjusting for all other factors in the table*

## Discussion

This is the first paper illustrates the smoking prevalence and its associated factors among a nationally representative sample of Malaysian elderly. The present findings showed that the prevalence of current smokers and ex-smokers were 15.2 and13.1 %, respectively, while the prevalence of current smokers was significantly higher in males as compared to females (28.1 % versus 2.9 %). The prevalence of smoking was lower than those reported in 2002 (39.1 %) by Lim et al. [10] and 19.8 % from national health and morbidity survey 2006 [11]. In addition, a reduction of approximately 11 % of male smokers had been observed between the present study and those reported by Lim et al. in 2006 [10]. The reduced smoking prevalence could be attributable to few government initiatives such as organisation of health campaigns to raise awareness on smoking hazards among the elderly and implementation of legislative measures such as prohibition of smoking in selected public areas.

The prevalence of smoking was also lower than the 28.1 % reported in Lebanon [29] but higher than the 11.5 % reported in Europe [30]. In terms of smoking prevalence by gender, the proportions of smoking by gender as observed in the present study were comparable to those reported by Kim et al. in 2013 [18] among the elderly in Korea (23.3 % in men; 3.9 % in women). There are few factors contributing to higher smoking prevalence in males than females. First, smoking has been a social milieu among Malaysian males. and men were less motivated the cease smoking as compared to their female counterparts [10], Second, a previous national study revealed that males initiated smoking at an earlier age and consumed more tobacco products, which would induce addiction toward nicotine [11]. The high nicotine level addiction might reduce the likelihood among males to cease smoking as compared to female elderly. However, further investigations should be undertaken to determine the contributing factors

On the other hand, no signification association was observed between age group and smoking status among elderly, and such findings were not in agreement with those reported by other investigators. Stotts and Smith reported that the proportion of smoking was 11.5 % higher among respondents aged 60–64 years than those aged 70–79 in Arkansas, USA [31]. Lugo and colleague also observed a significantly higher proportion of smoking among younger elderly (65–74 year-old, 13.4 %, 95 % CI 11.9-14.9 vs 75 years and above 8.2 %, 95 % CI 6.5-9.0) [30]. In addition, Honda [32] and Lim and colleague had also reported such similar findings. The present findings were not corroborated by the notion from previous literatures which posit that old age was always related to more disabilities, more sense of vulnerability and prone to have a greater likelihood of experiencing adverse health events from smoking, therefore older elderly were more receptive to public health messages and medical advice, and more likely to quit smoking few of the plausible reasons for the contrary findings as observed in the present study could be due to higher addiction level to nicotine among elderly smokers in Malaysia and less motivation to quit in view of not much benefit will be gained from deviating from current behaviour. Study to investigate the relationship between nicotine addiction level, perception toward smoking cessation and age groups among the elderly could be conducted to prove the above postulations.

Elderly people with higher educational attainment, which was significantly associated with smoking cessation in the univariable analysis, had also showed significant after the effects of other independent variables were controlled for. These findings were in line with the those reported by Tsai et al. in 2012 [33] who found that elderly with less than 6 years of formal education were more likely to smoke. Consistent with the finding reported by Kim and colleague [17], higher educational attainment was found to be a protecting factor for smoking. It was explicable that elderly with higher educational attainment may have better knowledge on smoking hazards and therefore less likely to smoke [34] Furthermore, less educated people may be less responsive and sensitive to health promotion, have less information on the health consequences of smoking and less access to cessation services [35, 36].

The present study did not find any association between marital status smoking and such findings were consistent with those reported by Lindstrom and colleague in a longitudinal study in Sweden [37]. Nonetheless, the present findings were contradictory to the findings reported by Peixoto et al. in 2005 who revealed that unmarried males were more likely to become smokers (aOR 1.87, 95 % CI 1.16-3.00) [38]. In addition, Tsai and colleague [33] and Lee & Kahendee [39] had also reported that elderly who lived with their spouses were more likely to cease smoking (aOR 10.21, 95 % CI 2.47–42.16). In contrast, being unmarried was found to be a factor associated with quitting smoking among adult Chinese males [40]. The contradict findings as observed in the present findings and those reported by Lindstrom and colleague in 2002 [37] and the Chinese males [40]. The finding were deviated from the ‘marriage protection’ and ‘marriage selection’ theories [41], which posit that emotional distress due to divorce causes divorcees to turn to smoking for relief. Moreover, these theories also suggested that married people tend to have more economic advantages, and therefore received more social and psychological supports which could make quitting smoking more likely. It is also possible that the healthier non smokers are more likely to get and stay married than those who are divorced as posited by the marriage selection theory. The contrary effects of marriage against smoking cessation across different populations suggested the presence of interactive nature of many variables and the role of culture in shaping smoking behaviour.

The present study revealed that the Malays and other Bumiputras were more like to smoke as compared to the Chinese and Indians and such findings were consistent with those been observed in the previous NHMS [11] and Global Adult Tobacco survey [12]. These findings underscore the importance to identify potential factors contributing to the association between ethnicity and smoking status for better planning and implementation of smoking control and prevention programmes in Malaysia.

One fifth of the elderly with monthly household income of more RM3000 were smokers and it was higher than their counterparts whose income were less than RM3000, however, the significant association between income and smoking was not observed in both univariate and multivariable analyses. These findings were contradicted to those reported by Huisman et al. [42] and Gilmore and colleague [43] in which they demonstrated that higher income level was a protective factor against smoking. On the other hand, Aekplakorn et al. [44], Cho et al. [45] and Lim et al. in 2010 [46] revealed that the likelihood of smoking was higher among those from lower income groups. The plausible reasons for the present findings could be due to increasing price of tobacco products since the last 10 years which had caused those from lower income brackets to cease smoking, while elderly with higher income may be still working at their old age and the stress induced by their occupations may drive them to smoking to relieve stress. In addition, the social mille in their working environment may also influence them to continue their smoking habits.

Respondents with known diabetes or hypercholesterolemia showed no significant impacts on smoking status. These findings were generally in line with the observations made by Honjo and colleague in 2010 [47] who studied among Japanese aged 40–59 years old and Tsai et al. [33] who investigated among Taiwanese aged 50–66. However, our results were not consistent with those reported by Kim et al. [48], who reported that in China and Europe, the most common reason for quitting is illness (ill-quitter effect). It was explicable that the diseases investigated in the current study (diabetes or hypercholesterolemia) were “silent” health conditions that did not cause functional impairments or traumatised conditions, therefore, it may not induce enough fear, motivation and higher levels of psychological distress to cause a change in smoking behaviour. On the other hand, the present study revealed that respondents with known hypertension were less likely to smoke, and this warrant further investigation on the dynamic relationship between diabetes, hypercholesterolemia, hypertension and smoking behaviour among Malaysian elderly to elucidate the real factor/s contributed to the finding in this study. Since the cross-sectional nature of the present findings had limited the inference of direct causal relationships between diabetes, hypercholesterolemia, hypertension and smoking.

The present study was not without limitations, as self-reporting of smoking status was subjected to under- or over-reporting of smoking status due to recall bias and the tendency of respondents to give socially desirable responses. Furthermore, the cross-sectional nature of the present study did not allow the establishment of cause-and-effect relationships. In addition, several factors which had been demonstrated to be associated with smoking from previous studies such as depression and stress [17, 49], dependence or addiction to nicotine [48], lack of more detailed data on smoking history and past attempts of quitting [32, 33] might have limited the analysis and interpretation of the present data. However, the large and representative sample as well as high response rate were the major strengths of the present studies.

## Conclusion

The high prevalence of smoking among the elderly in Malaysia, coupled with increased costs in medical care and an ageing population, may seriously impact the economy and increase the burden of healthcare in the country. Although Malaysia has signed and ratified the Framework Convention for Tobacco Control and the existence of tobacco control programmes and policy, the smoking prevalence is still holding high. Therefore, there is an urgent need to develop and implement aggressive and robust tobacco cessation programs which targeted at the older age groups to promote healthy ageing, especially in view of the epidemiological transition towards more chronic diseases and an increase in life expectancy in Malaysia.
